# Molecular and Environmental Determinants of Addictive Substances

**DOI:** 10.3390/biom14111406

**Published:** 2024-11-05

**Authors:** Małgorzata Lorek, Piotr Kamiński, Jędrzej Baszyński, Tadeusz Tadrowski, Edward Jacek Gorzelańczyk, Julia Feit, Natalia Kurhaluk, Alina Woźniak, Halina Tkaczenko

**Affiliations:** 1 Division of Ecology and Environmental Protection, Department of Medical Biology and Biochemistry, Faculty of Medicine, Collegium Medicum in Bydgoszcz, Nicolaus Copernicus University in Toruń, M. Skłodowska-Curie St. 9, PL 85-094 Bydgoszcz, Poland; malgorzatalorek1@gmail.com (M.L.); jedrzej.baszynski@cm.umk.pl (J.B.); 2 Department of Biotechnology, Institute of Biological Sciences, Faculty of Biological Sciences, University of Zielona Góra, Prof. Z. Szafran St. 1, PL 65-516 Zielona Góra, Poland; 3 Department of Dermatology and Venereology, Faculty of Medicine, Collegium Medicum in Bydgoszcz, Nicolaus Copernicus University in Toruń, M. Skłodowska-Curie St. 9, PL 85-094 Bydgoszcz, Poland; t.tadrowski@cm.umk.pl; 4 Institute of Philosophy, Kazimierz Wielki University in Bydgoszcz, M.K. Ogiński St. 16, PL 85-092 Bydgoszcz, Poland; medsystem@medsystem.com.pl; 5 Faculty of Mathematics and Computer Science, Adam Mickiewicz University in Poznań, Uniwersytet Poznański St., 4, PL 61-614 Poznań, Poland; 6 Primate Cardinal Stefan Wyszyński Provincial Hospital in Sieradz, Psychiatric Centre in Warta, Sieradzka St. 3, PL 98-290 Warta, Poland; 7 Department of Theoretical Foundations of Biomedical Sciences and Medical Computer Science, Faculty of Pharmacy, Collegium Medicum in Bydgoszcz, Nicolaus Copernicus University in Toruń, Jagiellońska St. 15, PL 85-067 Bydgoszcz, Poland; 8 Pallmed sp. z o.o., W. Roentgen St. 3, PL 85-796 Bydgoszcz, Poland; j.feit@domsueryder.org.pl; 9 Department of Animal Physiology, Institute of Biology, Pomeranian University in Słupsk, Arciszewski St. 22 B, PL 76-200 Słupsk, Poland; natalia.kurhaluk@upsl.edu.pl (N.K.); halina.tkaczenko@upsl.edu.pl (H.T.); 10 Department of Medical Biology and Biochemistry, Faculty of Medicine, Collegium Medicum in Bydgoszcz, Nicolaus Copernicus University in Toruń, M. Karłowicz St. 24, PL 85-092 Bydgoszcz, Poland; al1103@cm.umk.pl

**Keywords:** molecular determinants, addictive substances, chemical elements, genetic polymorphisms, determinants of addiction, opioid dependence, risk factors, addictive disorders

## Abstract

Knowledge about determinants of addiction in people taking addictive substances is poor and needs to be supplemented. The novelty of this paper consists in the analysis of innovative aspects of current research about relationships between determinants of addiction in Polish patients taking addictive substances and rare available data regarding the relationships between these factors from studies from recent years from other environments, mainly in Europe, and on the development of genetic determinants of physiological responses. We try to explain the role of the microelements Mn, Fe, Cu, Co, Zn, Cr, Ni, Tl, Se, Al, B, Mo, V, Sn, Sb, Ag, Sr, and Ba, the toxic metals Cd, Hg, As, and Pb, and the rare earth elements Sc, La, Ce, Pr, Eu, Gd, and Nd as factors that may shape the development of addiction to addictive substances or drugs. The interactions between factors (gene polymorphism, especially *ANKK1* (*TaqI A*), *ANKK1* (*Taq1 A-CT*), *DRD2* (*TaqI B*, *DRD2 Taq1 B-GA*, *DRD2 Taq1 B-AA*, *DRD2-141C Ins/Del*), and *OPRM1* (*A118G*)) in patients addicted to addictive substances and consumption of vegetables, consumption of dairy products, exposure to harmful factors, and their relationships with physiological responses, which confirm the importance of internal factors as determinants of addiction, are analyzed, taking into account gender and region. The innovation of this review is to show that the homozygous *TT* mutant of the *ANKK1 TaqI A* polymorphism *rs 1800497* may be a factor in increased risk of opioid dependence. We identify a variation in the functioning of the immune system in addicted patients from different environments as a result of the interaction of polymorphisms.

## 1. Introduction

Addictions as diseases of complex etiology, in which the interaction of socio-psychological, genetic, and environmental factors plays a fundamental role, are associated with the initiation of the use of addictive substances or drugs, including opioids, as well as the development of psychoactive substance use disorders (SUDs). Addiction (SUD) is a chronic, relapsing disease of the central nervous system that is complex in terms of etiology, molecular mechanisms, clinical course, and treatment. Interacting psychological, environmental, and genetic factors underlie this process.

The results of previous studies indicate that genetic factors influence addiction and relapse after treatment. The exposure to addictive substances or drugs causes marked changes in the expression of more than a hundred genes. However, it is important to discover those genes that play a key role in the mechanisms of action of these substances and the development of addiction. Genetic addiction research helps explain why some people are more sensitive to substance dependence [1,2]. Advances in genetic research among addicts may contribute to improving the effectiveness of addiction prevention and treatment. Knowledge about the environmental and genetic determinants of addiction in people taking addictive substances or drugs is poor and needs to be supplemented [2,3,4]. Thus, relationships between defense mechanisms and addiction need to be established.

Presumably, there is variation in the functioning of the immune system in people from different environments resulting from changes in genetic material. Changes in these processes, which are related to the impact of the environmental factors, may result from physiological mechanisms at the molecular level and from changes in the course of defense mechanisms. The impact of ions of various chemical elements combined in chemical bonds, as well as the impact of individual conditions and social factors, are very diverse and depend not only on the nature and composition of chemical bonds but also on the interaction of various risk factors, including mainly environmental and genetic factors. Therefore, each patient reacts to psychoactive substances in a way that depends on their individual physiological determinants and current environmental and social conditions and habits, in accordance with the WHO (2019, 2024) [3,4,5].

Addiction to addictive substances or drugs comprises a set of physiological, behavioral, and cognitive phenomena, among which the intake of a substance or a group of substances dominates over other phenomena that previously had a greater value for the patient. The main symptom of addiction is a strong, even overpowering desire to take a psychoactive substance (“hunger”, craving). A typical phenomenon is “addiction memory”, which consists of the rapid emergence of a full addiction syndrome even after many years of abstinence [1,2,3]. Substance dependence is a chronic, complex disease of the central nervous system in terms of etiology, molecular mechanisms, clinical course, and treatment. Not all people who use addictive substances or drugs become addicted. The propensity of individuals to initially try an addictive substance, to sustain taking it, and to eventually develop the progressive brain changes that characterize addiction, varies [5].

So far, the few studies in this area indicate that addictive substances or drugs are characterized by a large variety of chemical structures, different pharmacological effects, and different effects on the processes regulating motivational behavior and emotional functions [1,6,7]. According to the classification of environmental internal and external factors (WHO, International Statistical Classification of Diseases and Related Health Problems (ICD)) [1,3,4], addictive substance or drug dependence is defined as a mental and behavioral disorder caused by the use of these compounds. It distinguishes addictive substance dependence as addictive substance use disorder resulting from repeated or continuous use [4]. Criteria for addiction to a psychoactive substance assume that the patient is addicted when at least three situations occur during the year [3,8]: (1) a strong desire to take a given psychoactive substance or a compulsion to take it and subordination of life of this substance, (2) a lack of control over the intake of a substance, (3) taking more substances for a longer time than planned, (4) the occurrence of withdrawal symptoms after discontinuation of the substance or a reduction of the dose taken, (5) the occurrence of tolerance symptoms, (6) the need to take more and more of the substance, and (7) taking the substance despite the visible negative health effects and engaging in risky behavior.

The results of previous studies indicate that genetic factors influence addiction and relapse after treatment. Genetic addiction research helps explain why some people are more sensitive to substance dependence. Advances in genetic research among addicts may contribute to improving the effectiveness of addiction prevention and treatment [9,10,11,12,13,14]. However, knowledge about the environmental and genetic determinants of addiction in people taking addictive substances or drugs is still poor and needs to be supplemented. In order to isolate aspects related to the influence of environmental and genetic determinants of addiction in patients taking addictive substances, and in order to interpret this problem more appropriately, we comprehensively consider here the relationships between environmental and genetic determinants of addiction.

The novelty of this paper consists in the analysis of innovative aspects of current research about the relationships between environmental and genetic determinants of addictions in Polish patients taking addictive substances or drugs and rare available data regarding relationships between these factors from other environments, mainly in Europe, and the development of genetic determinants of physiological responses [15,16,17,18,19,20,21,22]. The totality of these processes in changing environmental conditions may indicate the causes of changes in the immune systems of addicts. Addictive substances or drugs taken over a long period are associated with activation of biochemical defense mechanisms, and they are related to genetic and environmental factors. The differences in the concentrations of chemical elements in patients addicted to addictive substances or drugs are determined here. We try to explain the role of the microelements Mn, Fe, Cu, Co, Zn, Cr, Ni, Tl, Se, Al, B, Mo, V, Sn, Sb, Ag, Sr, and Ba, the toxic metals Cd, Hg, As, and Pb, and the rare earth elements Sc, La, Ce, Pr, Eu, Gd, and Nd as factors influencing the development of addiction. Also, the interactions between environmental and genetic factors (gene polymorphisms, especially *ANKK1* (*TaqI A*), *ANKK1* (*Taq1 A-CT*), *DRD2* (*TaqI B*, *DRD2 Taq1 B-GA*, *DRD2 Taq1 B-AA*, *DRD2-141C Ins/Del*), and *OPRM1* (*A118G*)) in patients addicted to addictive substances and consumption of vegetables, consumption of dairy products, exposure to harmful factors, and their relationships with physiological responses, which confirm the importance of internal factors as determinants of opioid addiction, are analyzed, taking into account gender and region. Based on these analyses, the importance of these relationships between DNA variability and physiological responses to environmental stressors can be inferred.

The innovation of this review is also to show that the homozygous *TT* mutant of the *ANKK1 TaqI A* polymorphism *rs 1800497* may be a factor in increased risk of opioid dependence, and we try to explain a variation in the functioning of the immune system in addicted patients from different environments as a result of the interaction of polymorphisms. We also compare the changes in the functioning of organs related to the impact of the environment, which may result from epigenetic mechanisms. This review provides an understanding of the environmental factors that play a role in developing addiction to addictive substances or drugs, which in turn will enable taking action to reduce the risk of developing addiction to these substances. This review brings these innovative aspects in terms of missing data in the above scope and complements the existing knowledge in the discussed area of mutual relationships in patients with psychosomatic changes. Thanks to this, getting to know the environmental factors of addiction makes it possible to take actions aimed at reducing the risk of addiction. We check here which environmental factors in addicted patients increase susceptibility to addiction. It is possible to assess the risk of addiction, and, in case of an increased risk, it enables the selection of an alternative method of pharmacotherapy. Also, showing the type of gene polymorphism allows for the identification of a risk group for addiction in correlation with exposure to environmental factors. Thanks to this, it is possible to determine the genetic basis of addiction, which may enable the development of modern practices of pharmacotherapy for forecasting and preventing addiction. Therefore, these considerations may allow for suggested medical management in patients with greater sensitivity depending on the gene polymorphisms and genetic conditions relevant to the functioning of the immune system. Identification of polymorphisms responsible for increased susceptibility to addiction may improve the quality of pain treatment and reduce or eliminate the risk of dangerous side effects caused by addictive substances. So, this paper may be helpful in the diagnosis of psychosomatic changes and in the initiation of directed, more effective treatment of this group (pharmacological, hormonal). Physicians could be able to assess the risk of addiction in a given patient and, in case of an increased risk, choose alternative treatments.

## 2. Environmental Determinants of Addiction

Research so far indicates the existence of a relationship between environmental exposure and the occurrence of negative health effects and impacts on neurological functions [23,24,25,26]. There are not many reports on the influence of the environment on the development of SUD. Sussman et al. (2015) [27] found that increased exposure to environmental pollution may contribute to dysregulation of the dopamine release mechanism in the mesolimbic pathway, thus promoting compulsive use of addictive substances. This may be due to increased dopamine release in the nucleus accumbens septum (NAc) and the amygdala [27]. Environmental factors activate hormonal mechanisms of the hypothalamic–pituitary–adrenal axis, which, through mechanisms dependent on corticoliberin and glucocorticosteroid receptors, modulate the action of the dopaminergic system [28]. As a result, dysfunction of the dopaminergic system and dysregulation of motivational processes and the reward mechanism may occur, which may predispose a person to abuse of addictive substances [29].

Based on the findings of the International Classification of Mental and Behavioral Disorders (ICD) [4], we can formulate methods for the diagnostic determination of mental and behavioral disorders associated with the use of psychoactive substances. This classification includes disorders related to alcohol dependence, opioid and cannabis dependence, cannabinoid dependence, sedative, hypnotic, or anxiolytic dependence, cocaine dependence, stimulant dependence, including amphetamines, methamphetamine, or methcathinone, cathinone and hallucinogenic substances dependence, and nicotine dependence [4]. At the same time, specific environmental factors may modify the expression of predisposing genes associated with susceptibility to the development of addiction (SUD) [30]. Psychosomatic substance receptors are subject to epigenetic regulation, which is multi-stage and can be modulated at each stage. Epigenetic mechanisms are mainly DNA methylation, post-translational modifications of histones, and the action of micro-RNAs (miRNAs) [31]. Opioid receptor genes are rich in CpG islands (easily methylated sites), and this may affect their expression and modify the expression of the µ-receptor. The consequence of the methylation of CpG islands within the 5’ sequence of genes is the reduction or silencing of expression. Methylation affects the degree of chromatin condensation, which significantly reduces the availability of DNA for transcription factors [32,33,34]. The epigenetic regulation of genes changed by external or internal environmental factors encoding δ- and κ-receptors and endogenous opioid peptides has also been analyzed. The expression of genes encoding receptors and other genes involved in the development of addiction may be regulated by small non-coding micro-RNAs (mi-RNAs) [33]. Studies of epigenetic factors in the structures of the mesolimbic system prove that a short-term increase in the histone acetylation in the nucleus accumbens septum (NAc) correlates with the intensity of the behavioral response to addictive substances or drugs. Due to the countless combinations and overlapping modification possibilities, this is a very complicated process [35].

### 2.1. Exposure to Heavy Metals in the Diet

Exposure to heavy metals in the diet, especially in the early stages of life (childhood heavy metal intoxication), may be associated with the development of a proclivity to substance abuse. Animal studies have confirmed that chronic developmental dietary lead exposure alters µ-opioid receptor levels in the brain, and the main increase occurs during the juvenile and early adolescent period, while no changes or only minor changes appear in late adolescent and adult periods. In particular, the early developmental period distinguishes itself through higher engagement in reward-seeking behaviors in humans. Exposure to lead, based on animal models, may result in the disruption of the ontogeny of µ-opioid receptors, while alterations in µ-opioid receptor levels affect specific brain regions connected with addiction circuits and therefore could have implications for opioid abuse [36].

Another problem in this context is the connection between perturbations of the gut microbiome, microglial responses, and changes in the activation of the neuronal ensembles engaged in the process of intoxication and withdrawal in opioid use disorder. This implicates the critical role of the microbiome in modulating the brain’s response to particular substances. Consequently, microbiome depletion during antibiotic treatment may provoke serious alterations in sensitivity of the brain regions, which appear to play a decisive role in the course of addiction. On the other hand, the exposure to addictive substances perturbs the gut microbiome, leading to microbial dysbiosis. In addition, the gut may undergo a disruption resulting in a detrimental increase in its permeability. Finally, perturbations in the gut microbiome may stimulate microglia to release pro-inflammatory cytokines, thus inducing neuroinflammation. This process subsequently remodels synapses responsible for drug-related behaviors [37].

It is worrying that in various regions of the world, the content of heavy metals in foodstuffs still exceeds the limit values set by the WHO [3,4], and these values are considered harmless there. In many cases, accompanying concerns are expressed about the presence of such metals as cadmium, lead, arsenic, or chromium in drinking water. This problem may be connected to the improper application of metal-rich irrigation water, the poor management of industrial effluents, the improper usage of trace metal additives to poultry and fish feed, as well as the application of heavy-metal-containing pesticides and fertilizers. All of these factors may create a risk of transfer of heavy metals into the food chain, while health risks connected to their bioaccumulation besides carcinogenesis may also implicate immune system imbalance [38].

### 2.2. The Impact of Chemical Elements

Our own current studies [39,40,41,42] have shown significant differences in the concentration of most macroelements, microelements, toxic metals, and rare earth metals in the plasma of people addicted to opioids compared to controls. Our research has shown significant differences in the concentrations of ions of macroelements in the plasma of patients addicted to opioids compared to people from the control group. Opioids and other addictive substances caused significantly lower levels of P and higher levels of Na, K, and Ca than those in the controls [39,40,41,42]. An increase in Na, K, and Ca concentration and a decrease in P concentration in the plasma of people addicted to opioids may be the result of changes in the metabolism of these elements under the influence of opioids. Radovanovic et al. (2012) [43] found hyperkalemia in a patient addicted to heroin. These authors suggest that chronic opioid use often leads to organ damage, acute renal failure, rhabdomyolysis, and electrolyte disturbances. For example, the U.S. Food and Drug Administration (FDA) points to the relationship between methadone hydrochloride and increased blood potassium levels. Research has analyzed how many people taking methadone hydrochloride have elevated blood K levels. In 2021, 3667 people reported adverse reactions while taking methadone hydrochloride; among them, 13 people had elevated blood K levels [44]. Most data, however, show decreased blood K and Ca levels in opioid-dependent individuals. Studies by Elnimr et al. (1996) [45] showed a decrease in the concentration of K and Ca in the plasma of people addicted to opioids. It has been proven that K and Ca concentrations decrease with the duration of addiction, although the K concentration after one year of addiction (371 µg*L^−1^) was higher than in the controls (337 µg*L^−1^) [45]. Studies by Divsalar et al. (2010) [46] and Afarinesh et al. (2014) [47] showed that the plasma K concentration was lower in the group of opium addicts compared to the control. Plasma Ca levels were reduced in the opium-addicted group compared to the controls [46,47].

In our current studies [39,40,41], a significant, positive, and very strong correlation between Ca and Na concentrations and a positive, strong correlation between K and Mg concentrations were found in patients, which suggests that with increasing Ca, the average Na concentrations increase, and with the increase in K concentrations, Mg increases. There was also a significant positive correlation between K ions and lipoperoxidation intensity (MDA) and a positive correlation between Ca ions and lipoperoxidation intensity (MDA). The increase in K and Ca is accompanied by an increase in the average MDA concentrations. In the blood, it is an unfavorable signal, because it indicates active peroxidation of polyunsaturated fatty acids and the presence of oxidative stress. Our studies also showed that people addicted to opioids have significantly lower levels of phosphorus than controls. Decreased plasma P levels may be the result of malabsorption (vitamin D deficiency), metabolic disorders, opioid intake, or insufficient phosphorus intake in the diet. Our studies [39,40,41] showed that people addicted to opioids used vitamin D3 less frequently (6.8%) than people in the control group (20%) and consumed vegetables less often (34%) compared to the control group (58%).

Elnimr et al. (1996) [45] found a decrease in the concentration of phosphorus in the plasma of people addicted to opioids. They proved that plasma P concentration decreased with the duration of addiction. In the control, the concentration of phosphorus was 127 µg*L^−1^; in addicts, after a year of addiction, it was 92 µg*L^−1^; after 4 years, it was 32 µg*L^−1^; and after 6 years, it was 11 µg*L^−1^ [45]. A retrospective study compared plasma P levels in heroin addicts and healthy subjects. There were no differences in P concentrations between heroin addicts and healthy people [48]. In our studies, significant positive correlations between P, Na, and Mg were shown in patients. There was also a significant positive correlation between P and bilirubin, which confirms the positive effect of P on the functioning of the second line of antioxidant defense. We also showed that people addicted to opioids have a significantly higher level of micronutrients compared to healthy people. The results may indicate a higher burden of trace elements in patients, which may have an impact on the development of opioid dependence [39,40,41].

At the same time, the importance of chemical combinations of Co ions for shaping the system in reacting to psychoactive compounds and impairing the reaction at the molecular level in the situation of addiction to opioids and addictive substances or drugs should be emphasized. Cobalt in excess reduces the amount of neurotransmitters (dopamine, serotonin, norepinephrine) in the central nervous system, induces ROS formation, and lowers the level of reduced glutathione [49]. In the studies by Adachi et al. (2011) [50], cobalt chloride inhibited the activity of extracellular SOD in the pericytes of the retina, resulting in increased ROS production, caspase activation, and DNA fragmentation. There are no studies comparing Co levels in opioid-dependent and healthy individuals. Only in our studies [39,40,41] did we find that a significant positive correlation of Co and Cr occurs in patients. We also noted a significantly increased concentration of Zn in the plasma of people addicted to opioids compared to the controls. However, Elnimr et al. (1996) [45] and Martinez et al. (1990) [51] found a significant decrease in the concentration of Zn in the blood of heroin addicts compared to healthy people. The decrease in the Zn concentration in the blood resulted from the increased excretion of Zn in the urine or from nutritional deficiencies.

Different research shows that Zn ions interact in the neuronal pathways with receptors for NMDA glutamate. Zinc inhibits the flow of ions in the NMDA glutamate receptors and reduces the affinity of glycine for this receptor, which is necessary in the process of its stimulation. These receptors are involved in the pathogenesis of addiction [52,53]. In addition, NMDA glutamate receptors can affect dopamine transmission [54]. Zinc also affects other ionotropic glutamate receptors, such as AMPA, which are activated faster than NMDA and affect synaptic plasticity more intensively [55]. These data suggest that the disruption of Zn homeostasis may have an impact on opioid dependence. Zinc in complexes with chelators may reduce morphine withdrawal symptoms by enhancing the activity of the opioid system. Dursun et al. (1995) [56] showed that Zn deficiency reduces the antinociceptive (analgesic) effect of morphine in rats, and these changes are dose- and time-dependent. Zinc may be required for the analgesic effect of morphine [55]. Ciubotariu and Nechifor (2007) [57] tested whether Zn supplementation affects the severity of morphine addiction. A reduction in the severity of morphine dependence was confirmed, and the effect was dose-dependent [57]. Mesbahzadeh et al. (2019) [58] showed that Zn influences the enhancement of conditioned place preference (CPP) induced by morphine through the serotonergic and dopaminergic systems. CPP occurs when a subject prefers one place because the location has been previously linked to satisfying feelings [58]. Zinc, by affecting the expression of opioid receptors, the affinity of morphine to its receptors, and the activity of glutamate receptors (NMDA, AMPA), may have a significant impact on the nervous system pathways associated with opioid addiction. Low levels of Zn in people using opioids may contribute to the development of addiction and reduce the effectiveness and extend the duration of treatment. On the other hand, exposure to high levels of Zn causes a focus of neurological deficits, which may affect the functions of the reward system [59]. Our studies [39,40,41] showed a significant positive correlation between Zn and Ca, a significant positive correlation between the concentration of Zn and retinol, and a negative correlation between Zn and uric acid. These studies indicate that zinc may inhibit the second line of antioxidant defense.

When discussing the relationship between the activity of chemical compounds’ connections and addiction to opioids and addictive substances or drugs, one should also take into account the important role of redox-active micronutrients in shaping the system reacting to psychoactive compounds and impairing reactions at the molecular level in the case of addiction to opioids and addictive substances or drugs. Chromium has an anxiolytic and antidepressant effect, and its effect is due to serotonergic, noradrenergic, glutamatergic, and dopaminergic transmission [60]. Ciubotariu et al. (2018) [61] found the effect of Cr in alleviating the effects of morphine withdrawal and reducing the severity of morphine use. In our current research [39,40,41], it was shown that this effect comes from Cr in positive people, and it was significantly higher than in healthy people. Positive correlations of Cr with Na, Sc, and V were also found. Also, thallium has an important meaning for opioid addicts. Molavi et al. (2020) [62] studied the concentration of thallium in the urine, blood, and hair of people using illicit opioids, as well as the clinical symptoms of thallotoxicosis compared with the controls. They showed that thallium concentrations in the urine, blood, and hair of illicit opioid users were significantly higher than those of non-users. This may have been due to the use of Tl-contaminated opioids. Long-term use of illicit opioids can lead to Tl exposure [62]. Our studies [39,40,41] showed that thallium in the plasma of addicts was significantly higher than in healthy people, and there was no correlation between thallium and enzymatic and non-enzymatic factors of antioxidant defense in the group of patients. A significant positive correlation was found between Tl and Sb.

In considering the mutual relationships between the activity of chemical compounds and addiction to addictive substances or drugs, similar antagonistic interactions very often occur with other micronutrients, which cause serious physiological consequences. They are visible in the changes in the course of pro-antioxidant reactions at the cellular level, and this generates changes in the system reacting to psychoactive compounds and various impairments of reactions at the molecular level in the situation of addiction. For example, Wong et al. (1964) [63] reported cases of boric acid poisoning in neonates who ingested 4.5–14 g of this substance (tremors, restlessness, convulsions, coma). Histological examination showed cerebral and meningeal edema, perivascular hemorrhage, and intravascular thrombosis [63]. Our current research in this subject [39,40,41] showed that the concentration of boron in the plasma of addicts was significantly higher than in healthy people. A significant negative correlation between B and uric acid was found among opioid addicts, which proves the inhibitory effect of this element on the second line of antioxidant defense. Also, molybdenum in higher concentrations may change the course of pro-antioxidant reactions at the cellular level, and this generates changes in the system reacting to psychoactive compounds and various impairments of reactions at the molecular level in the situation of addiction.

Helaly et al. (2018) [64] found inflammation and degeneration of neurocytes in the cerebral cortex and hippocampus of rats receiving 30 mg of molybdenum for 30 days [64]. In our current research on this subject [39,40,41], plasma Mo concentration was higher in addicts, and, in patients, Mo correlated most strongly with Mn. Similarly, strontium can interact with cellular secondary messenger systems and transporter systems that normally interact with calcium. Therefore, it can also affect synaptic transmissions [65]. No studies have been found that indicate the neurotoxic effect of strontium in humans following dietary exposure. Only Johnson et al. (1968) [66] found hind limb paralysis in rats fed strontium at 565 mg*kg^−1^ daily for 43 days. The paralysis could be related to abnormal Ca levels in the muscles or nerves. Our studies on this subject [39,40,41] showed that the Sr concentration in the plasma of addicts was significantly higher than in healthy people. A significant positive correlation between Sr and Cu concentrations was observed in patients. On the other hand, toxicity associated with Sr excess is related to dysregulation of the transformations and functions of Cu, Ca, P, and Co [67,68]. There was also a negative correlation between Sr and catalase activity and a negative correlation between Sr and glutathione levels. These results indicate the inhibitory effect of strontium on the functioning of the first and second lines of antioxidant defense.

Little information was found on the association of cobalt, nickel, boron, molybdenum, vanadium, antimony, silver, tin, and strontium with opioid and addictive substance or drug addiction. Their role in shaping the reaction towards psychoactive compounds and the impairment of reactions at the molecular level in the situation of addiction to opioids and addictive substances or drugs should be emphasized here. Our studies [39,40,41] showed that people addicted to opioids had significantly higher concentrations of Cd, Pb, As, and Hg in the plasma compared to healthy people. This indicates a higher burden of toxic metals in patients, which may affect the development of opioid dependence.

Cadmium in high concentrations in tobacco products may alter the stimulant properties of morphine. Chronic Cd exposure increases alcohol and cocaine consumption [69]. Studies have shown that in animals chronically exposed to Cd, the effect of morphine is weakened, locomotor activity decreases when morphine is administered once, and locomotor activity increases when the drug is administered chronically [70]. Miller and Nation (1997) [71] put half of the rats on a cadmium chloride diet. They then administered morphine sulfate intraperitoneally, in the next study intracerebroventricularly, and in the next study subcutaneously. They showed a decrease in the reinforcing properties of morphine and fentanyl in rats receiving cadmium in their diet. Cd poisoning can lead to the abnormal functioning of pathways related to the limbic system, and it blocks the voltage-dependent channels for NMDA glutamate and thus reduces the stimulated release of dopamine and binds to D2 dopaminergic receptors in a competitive place for dopamine. Cd acts as a competitive antagonist of µ-opioid receptors. This may be related to Cd neurotoxicity and the impact on the development of opioid addiction [52,71,72]. In our research on this subject [39,40,41], a significant, negative correlation between Cd and retinol was found in patients. Positive correlations were also found between Cd and Na, Sc, Ca, and Cu.

Considering the participation of micronutrient ions in interactions at the cellular level in the case of addiction to addictive substances or drugs, finally, it is important to consider the importance of those that participate in the transformations that also occur during neurotoxic changes. These include ion-active red-ox chemical connections of toxic metals that cross the blood–brain barrier. Among them, the most important are lead, arsenic, and mercury. Lead impairs the functioning of neural pathways related to the pharmacodynamics of opioids, alters dopamine metabolism and the expression of dopamine receptors, and causes neuritis and increases tolerance [52]. Research to date suggests that lead affects neural pathways involved in the development of opioid addiction. Exposure to lead increases dopaminergic activity and is associated with attention deficits, Alzheimer’s disease, and increased drug sensitivity [73]. Lead affects purinergic and dopaminergic transmission and activates microglial cells. Lead increases the expression of purinergic receptors. Excessive activation of P2X purinergic receptors leads to inflammation, and microglia and astroglia are activated. In a state of chronic inflammation in the nervous system, microglial cells remain activated for a long time, releasing cytokines and neurotoxic molecules. In studies conducted by Listos et al. (2013) [74], induction of inflammation in rats through exposure to lead acetate was responsible for increased tolerance to morphine.

It is impossible to distinguish the effects of heavy metal ions that come from opioid impurities from other sources of heavy metals. In our own current studies [39,40,41,42], patients were on substitution treatment with methadone. Illegal opioids and addictive substances may be contaminated with metals, e.g., lead, thallium, and arsenic, which can cause clinical symptoms of poisoning in addicts taking these drugs. Wong et al. (2020) [75] reported cases of lead poisoning in patients who used illicit opiates in Australia after the Victorian Department of Health issued a health alert in 2018 after four cases of lead poisoning related to illicit opium use in Melbourne [75]. Opioid and addictive substance users may exhibit symptoms related to the central or peripheral nervous system, gastrointestinal complications, and anemia. In such cases, lead poisoning should be suspected, and chelation therapy should be started as soon as possible [76]. Cases of arsenic (As_2_O_3_) poisoning in people using illicit, contaminated opioids are described in the literature. These reports refer to the 1980s [77,78]. Krokodil (“heroin of the poor”) is the street name given to a homemade drug known as a cheap substitute for heroin. Krokodil use began in Russia and Ukraine. Krokodil is produced from codeine tablets under clandestine and unsanitary conditions. Krokodil contains large amounts of phosphorus, iodine, metals, such as iron, zinc, lead, and antimony, organic compounds, solvents (gasoline), and paint thinner, which are used to extract codeine [79,80].

Inflammation caused by lead may affect the processes of chronic morphine addiction. People taking illicit opioids may be at additional risk of poisoning. Shabani et al. (2020) [81] studied Pb concentration in opiate-dependent patients with unexplained, refractory abdominal pain. A correlation has been shown between Pb toxicity and abdominal pain, constipation, and paresthesia. Researchers suggest that lead toxicity should be considered in the differential diagnosis of severe and persistent abdominal pain in patients presenting to an outpatient clinic if they are addicted to illicit opiates. Illicit opioids may be contaminated with heavy metals [81]. Ghane et al. (2018) [82] reported a “lead poisoning epidemic” among opium users in Iran. Pb-contaminated opium and heroin are a global threat [82]. Our current research [39,40,41] showed that in people addicted to opioids, there is a significant positive correlation between Pb and Ba and a negative one between the level of Pb and K.

## 3. Risk Factors for Opioids and Addictive Substances

Opioids is the term used for all substances that act on opioid receptors, i.e., natural opioids (opiates, opium alkaloids from the opium poppy, *Papaver somniferum*), their semi-synthetic and synthetic analogues, and endogenous compounds synthesized physiologically. Natural opioids are morphine and codeine, and their semi-synthetic analogues are hydromorphone, oxycodone, hydrocodone, and heroin. Synthetic opioids include fentanyl, methadone, pethidine (meperidine), buprenorphine (as a semi-synthetic thebaine/oripavine derivative), tramadol, levorphanol, propoxyphene, and pentazocine. Endogenous opioid compounds include enkephalins, endorphins, dynorphins, and endomorphins [1,83]. Opioids are among the most addictive substances known to humans [6]. Repeated use of opioids causes a series of neuroadaptive changes in various neural circuits in the brain that are related to motivation, memory, and behavioral control. The result is an increased and sustained reward associated with opioid use and a concomitant decreased reward associated with natural rewards encountered in everyday life [1,7].

**Figure 1:**
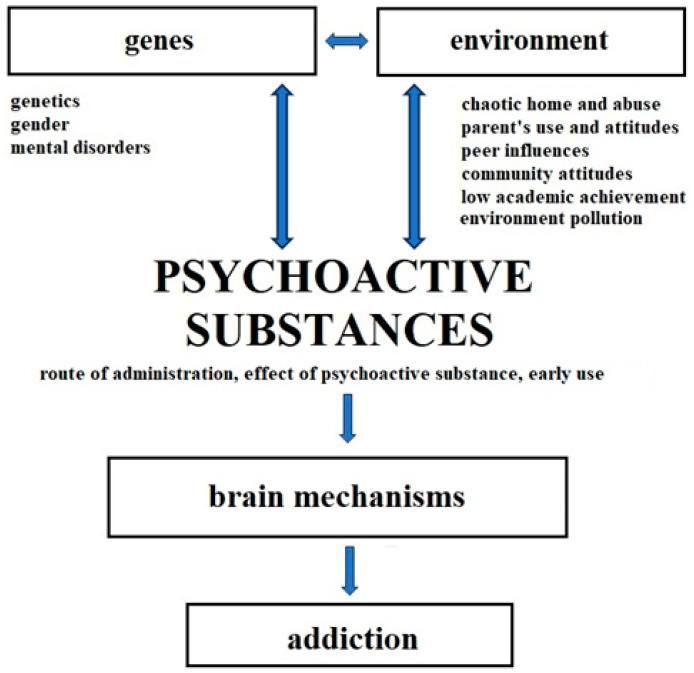
Risk factors for psychoactive substance use and addiction (modified after Volkov (2020) [88]).

Social risk factors include low economic status, unemployment, living in a poor district, being surrounded by people who have contact with addictive substances or drugs, an incomplete family, lack of support among loved ones, and the presence of addicts in the family [8]. Addiction factors include personality factors (low self-esteem, lack of ability to cope with stress and negative emotions, lack of problem-solving skills, and psychosocial deficits in interpersonal skills) [84,85]. An important role in the susceptibility to the development of addiction is played by the spectrum of obsessive–compulsive disorders. The tendency towards impulsive behavior consists of impaired drive control with an increased tendency towards risky behavior, and it is associated with the entire spectrum of the problem behaviors [86]. The tendency towards compulsive behavior (persistent repetition of behavior that is not appropriate to a given situation) is associated with the addiction process [87]. It should be emphasized that the influence of ions of various elements in chemical combinations and the influence of individual, social, or personality factors is very diverse and depends not only on the nature and composition of the chemical bond but also on the mutual interactions of various risk factors. Thus, a given patient reacts to psychoactive substances in a way that depends on their current conditions and physiological, environmental, habitual, and social predispositions (e.g., Table 1, Figure 1).

Different studies have shown that neurobiological and connected neuropsychological risk factors contribute to the development of addiction to illicit addictive substances or drugs are also essential for a proper understanding of the effects of environmental and genetic factors in patients taking addictive substances or drugs. These stressors can be defined as social factors, personality factors, biological factors (mental disorder), and individual factors (age, gender) [8]. Neurobiological and neuropsychological factors of addiction to addictive substances or drugs refer to the influence of the environment, i.e., so-called socialization patterns (family environment, peer environment, school environment, place of residence) and the so-called social context [89]. For example, 60% of the American population has had exposure to illicit substances at least once. Only a small percentage develop a clinically significant dependence syndrome during their lifetime, and, even in cases of highly addictive opioids, this likelihood ranges from 8 to 12% in the treatment of chronic pain. Furthermore, 20–30% of patients who take prescription opioids for chronic pain later abuse them, and 80% of those who use heroin first started abusing prescription opioids [5].

The research so far shows a significant link between mental disorders and addiction. The prevalence of substance dependence disorders is increased in adults with depression, schizophrenia, affective disorders, and antisocial and borderline personality disorders and in people with behavioral addictions. Nearly half of patients diagnosed with schizophrenia have a co-occurring substance use disorder [90]. Two types of associations between comorbid mental disorders and addiction are considered: whether substance abuse causes a higher risk of developing mental disorders and whether addiction and mental disorders are caused by the same factors (genetic susceptibility, stress). Many studies have confirmed that affective and anxiety disorders occur earlier than substance abuse and addiction [91]. The compulsion to undertake certain activities that may lead to addiction usually appears at a young age. Adolescents and young adults are more likely to suffer from a substance use disorder than older adults. The use of addictive substances or drugs by adolescents increases the risk of addiction in the group of adults. People with a history of drug abuse are more likely to become addicted when treated with opioid painkillers in adulthood. An earlier onset of substance use may cause neurobiological and neuroplastic changes in the rapidly developing brain [92,93]. According to the European Monitoring Center for Drugs and Drug Addiction (EMCDDA), in 2019, in the European Union, the average age of people at the time of first opioid use was 23, and the average age upon starting treatment for addiction for the first time was 36. Among opioid addicts entering treatment, 19% are women and 81% are men [94]. Studies so far indicate a higher incidence of SUD in men than in women [95,96].

As we present above, risk factors for addiction include individual, social, personality, and biological factors [8]. In research conducted by our team on this subject [39,40,41,42], it was found that the strongest factors predisposing people to the occurrence and development of opioid and addictive substance or drug addiction were male gender (odds ratio OR = 7.5), place of residence in cities (OR = 2.8), employment (type of work) (OR = 19.9), and family history of drug and opioid addiction (OR = 6.0). In these studies, in patients addicted to opioids, the majority were men (82%). Male gender is an important individual factor associated with the occurrence of opioid dependence. According to the WHO and the United Nations Office on Drugs and Crime (UNODC), women using addictive substances or drugs become addicted faster and while using lower doses than men, and they account for 33% of drug users (cannabis, cocaine, amphetamine, opioids). Furthermore, one in six addicts treated is a woman [1,97]. In the studies by Serdarević et al. (2017) [98], opioid drugs were used more often by women than by men. Women are more likely to experience chronic pain, and men are less likely to seek help from healthcare [98,99]. The results of our team [39,41] show that the largest number of people (90%) addicted to opioids and addictive substances or drugs live in cities with more than 10,000 inhabitants, which are largely burdened by the use of illegal addictive substances or drugs [100].

Our current research has shown that neuropsychological risk factors for addiction include low economic status and unemployment [39,40,41]. People addicted to addictive substances or drugs more often performed physical work (59%) than people from the control group, among whom mental work was dominant (68%). Furthermore, 32% of addicts had the status of an unemployed person or a pensioner. More opioid addicts (8%) had high exposure to harmful factors at work compared to the control group (7%). More addicts (6%), compared to the control group (0%), were exposed to heavy metals at work [39,40,41]. Social risk factors for addiction include being surrounded by people who have contact with addictive substances or drugs and the presence of addicts in the family [8]. Our research [39,40,41] has shown that among opioid addicts, addiction to drugs and opioid drugs is more often present in the family among fathers (3%), siblings (7%), and grandmothers/grandparents (1.4%) of opioid addicts. Opioid use disorders run in families [101,102,103,104]. Among the families of healthy people, addiction to drugs and medicines was not observed, and alcohol dependence was observed less often. Neurobiological factors and mental disorders also have a significant impact on addiction. Thus, among opioid addicts, depressive disorders were reported more often (42.5%) than in the controls (10%) [39,40,41], and, compared to healthy people, a much larger proportion of substance addicts suffer from another mental disorder. Twin studies suggest that this comorbidity may be due to a common genetic background that determines susceptibility to addiction and other mental disorders [105].

### Pathomechanism of Opioid Addiction

Opioid addiction is characterized by dysregulation of the systems responsible for reward mechanisms and motivational processes in the brain. Tolerance to an addictive substance develops as well as the habit of abuse and an uncontrollable desire to take it. The results of frequent relapses in using opioids, repeated episodes of intoxication, and withdrawal states are changes in the structure and functioning of the brain. There are neuroplastic changes in synapses (e.g., changes in the stability of dendritic spines) in the reward system, modifications in neuronal networks, disorders in neurochemical systems, and changes in the balance between various neurotransmitter systems [40,106,107]. The changes in the neuronal circuits are similar to the changes observed in learning and memory. This makes the risk of relapse in the case of opioids high even after long periods of abstinence. Neuroplastic changes are considered to be the molecular basis of addiction [40,106,108].

All addictive substances activate the brain’s reward system, causing a spike in dopamine release. In humans, rewarding effects require the activation of dopamine neurons in the ventral tegmental area (VTA) and the release of dopamine and endogenous opioids in the anterior ventral striatum, the nucleus accumbens (NAc). Dopamine is directly responsible for the exciting rush that fuels the urge to use opioids, and it plays a key role in the development of addiction itself. Taking an addictive substance that causes changes in the dopamine levels in the brain is associated with feelings of pleasure, which provide positive reinforcement [109]. Its repeated use causes sensitization to extracellular levels of dopamine in the NAc. Dopamine binds to receptors belonging to two families: the D1 family (D1, D5) or the D2 family (D2, D3, D4). They are metabotropic receptors with seven transmembrane segments. The D1 family of receptors couples to Gs protein, which stimulates cyclic adenosine monophosphate cAMP signaling. Receptors from the D2 family have the opposite effect; they are coupled with the Gi protein, they inhibit adenylate cyclase, and they activate potassium channels, thus causing hyperpolarization and the inhibition of neuronal function. They also inhibit the action of calcium channels, which affects the release of neurotransmitters. The D2 receptors are both presynaptic, which means they regulate the amount of dopamine secreted, and postsynaptic, which means they have a high affinity for this substance. Postsynaptic D2 receptors are activated through the slow firing of neurons. D1 receptors are postsynaptic receptors and require a higher concentration of dopamine to activate [110,111].

**Figure 2:**
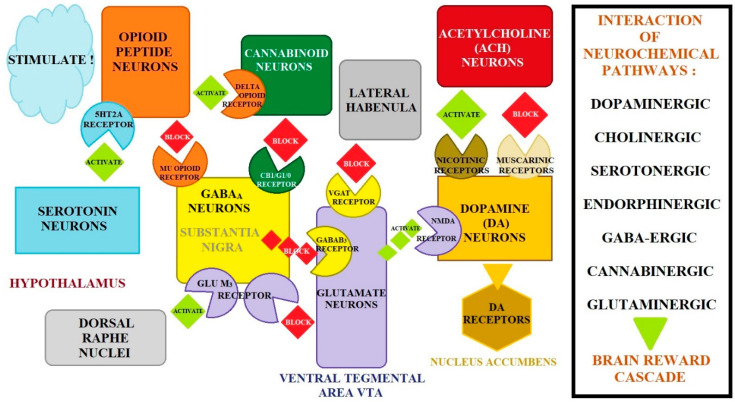
Brain reward cascade (modified after Moran et al. (2021) [113]).

An important feature of dopaminergic neurons of the mesolimbic system is that dopamine works together with other neurotransmitters on the basis of co-transmission. Neurons are modulated by inhibitory and excitatory neurotransmitter systems. The interaction of a minimum of seven neurochemical pathways (dopaminergic, cholinergic, serotonergic, endorphinergic, GABAergic, cannabinergic, and glutaminergic) together constitutes the brain reward cascade (Figure 2). The stimulating effect is exerted mainly by cholinergic neurons (via nicotinic receptors) and serotonergic neurons (via 5-HT3 receptors). The inhibitory effect is exerted by GABAergic neurons, which are additionally controlled by opioid and glutamatergic receptors. The activation of glutamatergic receptors enhances the inhibitory effect of GABA, and stimulation of µ-opioid receptors leads to inhibition of GABAergic neurons in the VTA, which in turn releases dopaminergic neurons from their inhibitory effects in the NAc [112,113]. Other brain structures are also involved in the mechanism of opioid addiction, including the thalamus, the prefrontal cortex, the cingulate cortex, the amygdala, and the hippocampus. Sensory information passes through the thalamus; it is characterized by a large number of opioid receptors, including, in particular, the µ-receptor. The prefrontal cortex plays an important role in judgment, planning, and other executive functions, including controlling opioid “cravings”. The cingulate cortex controls opioid “craving” as well as anxiety and mood. Amygdala syndrome is responsible for emotional behavior, memory, anxiety levels, and aggression [114].

In the pathogenesis of addictions, an important role is played by the hippocampus, a structure associated with declarative and spatial memory, the accumulation of memories, and the learning process. It receives information from the septal nuclei (cholinergic projections), from the VTA (dopaminergic projections), from the raphe nuclei (serotonergic projections), from the locus coeruleus (noradrenergic projections). Hippocampal stimulation intensifies glutamatergic transmission, indirectly increasing the release of dopamine in the NAc. In case of opioids, unlike other drugs, withdrawal leads to better learning and memory dependent on the hippocampus. The hippocampus is characterized by high synaptic plasticity, which facilitates the development of learning the drug context, which may facilitate the return to addiction. This structure plays an important role in the mechanism of positive reinforcement, which perpetuates the reaction and increases the probability of its repetition in order to obtain contact with a specific stimulus. Opioids modulate synaptic transmission and plasticity in the hippocampus. They significantly change glutamatergic transmission, affect neurogenesis, the stability of dendritic trees and long-term synaptic strengthening [106,115].

## 4. Genetic Determinants of Addiction

Considering the mutual relationships between the action of ions of chemical compounds and addiction to addictive substances or drugs, it is finally necessary to analyze the role of epigenetic processes and genotoxicity in shaping changes in the direction of the following reactions, especially at the molecular level, in patients addicted to addictive substances or drugs taking strong substitution drugs. During addiction, mutual reactions of antagonisms and synergies take place there, guided by pro-antioxidant processes and depending on the presence of gene polymorphisms. This causes serious physiological consequences. They manifest in changes (dysfunctions) in the course of pro-oxidative reactions at the cellular level, and this generates changes in the system in response to psychoactive compounds and various types of impaired reactions at the molecular level in the situation of addiction or SUD. This is very important, especially when we are dealing with long-term addiction, and it is a source of often irreversible changes in the nervous system. It is thus important that the share of genetic factors in susceptibility to the development of drug addiction is at the level of 40–60%. There is evidence of a common genetic susceptibility to two or more addictive substances or drugs: opioids, cannabis products, and stimulants [116].

In order to determine the contribution of genetic factors to the etiopathogenesis of a given disease, the results of genetic tests conducted on families, twins, and siblings given up for adoption have been analyzed. In family genetic studies, the frequency of disorders among relatives of the patient is compared with the frequency of their occurrence in the general population [117]. Twin studies compare the phenotypic similarities between monozygotic twins, who share identical genes, and dizygotic twins, who have, on average, half of the same genes, in terms of disease or quantitative traits [118]. Studies conducted by Merikangas et al. (1998) [101] involved 87 opioid addicts and 61 healthy and 1267 adult first-degree relatives. Relatives of opioid-dependent probands have been shown to have a 10-fold increased risk of developing opioid dependence compared to controls [101]. In research by Tsuang et al. (1998) [102], 3372 pairs of male twins participated, of which 1874 were monozygotic and 1498 were dizygotic. Drug dependence was found to be prominent in 9.5% of the study population. The co-occurrence of drug abuse across different categories (marijuana, sedatives, heroin) was also studied within the individual as well as twins to determine how genetic and environmental factors contribute to the development of addiction.

Genetic factors were found to have the greatest influence on the development of heroin addiction (38%) compared to the influence of these factors on the development of addiction to other substances tested [102]. The results of studies of families, twins, and adoptive families revealed that genetic factors have an impact on the development of addiction, but the interaction between genetic and environmental factors plays a fundamental role in the etiology [102,118,119,120]. The risk of developing an addiction related to the influence of genes depends on the type of substance taken; it is strongest in patients addicted to opioids (cocaine, alcohol) and weakest in the case of hallucinogens and amphetamines [121,122]. Research shows that opioid use disorders run in families [101,102,103,104].

### 4.1. Genetic Addiction Risk Scores

The first patented clinical test in Europe and the USA for predicting vulnerability to pain and various addictive behaviors (reward deficiency syndrome) was the Genetic Addiction Risk Score (GARS) test, i.e., the risks of genetic dependence (Table 2). The brain reward cascade includes the interaction of genes and neurotransmitters that control the release of dopamine (Figure 2). Functional differences within the brain reward cascade (genetic or epigenetic) may predispose individuals to addictive behaviors [113,123].

Progress in genomics and research on single nucleotide polymorphisms (SNPs) of genes has allowed for the selection of key genetic variants that may be a factor in disease susceptibility. Various methods for searching for associations of gene polymorphisms with specific diseases have been described. Association studies of candidate genes are population studies that consist of comparing the frequency of specific alleles and genotypes of polymorphisms of a given gene among unrelated people with the frequency of the same alleles and genotypes in healthy people [125,126]. These studies looked at candidate genes that were hypothesized to be involved in the disease. Genetic variants that determine susceptibility to the development of opioid addiction are associated with genes encoding active enzymatic, receptor, and transport proteins, which include enzymes metabolizing xenobiotics (cytochrome P-450 enzymes), enzymes metabolizing neurotransmitters (dopamine β-hydroxylase DβH, monoamine oxidase MAO), neurotransmitter receptors (dopamine receptors, primarily D2 receptor), receptors through which opioids exert their effects on the body (opioid receptors), and neurotransmitter transporters (dopamine DAT1, serotonin 5-HTTLPR transporters) [127]. Research has revealed the importance of polymorphisms in the genes of dopaminergic pathways and µ-opioid receptor in the development of opioid use disorders [42,128,129,130].

### 4.2. TaqI A Polymorphism (rs 1800497) of ANKK1 Gene

**Figure 3:**
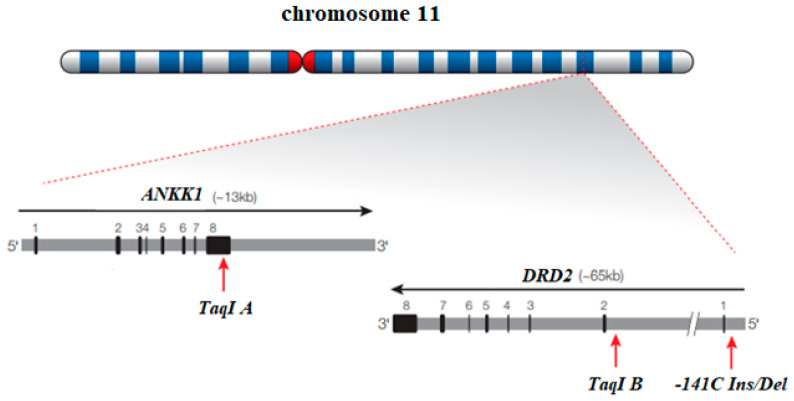
The structure of the *ANKK1* gene and the locations of the SNPs: *Taq1 A* in the *ANKK1* gene, the structure of the *DRD2* gene, and the location of two SNPs, *Taq1 B* and *-141C Ins/Del*, in the *DRD2* gene (modified after Zahari et al. (2011) [136]).

The human *ANKK1* gene (ankyrin repeat and kinase domain containing 1) is located on chromosome 11 (11q23.2), and it covers an area of 13 kb. It consists of eight exons. It encodes a protein that contains 11 ankyrin repeats and a serine–threonine kinase domain, which consists of 765 amino acids and belongs to signaling proteins [131,132]. This protein is involved in data transformation processes in the central nervous system. There are at least three protein isoforms: ANKK1 containing kinase RIP (receptor-interacting protein) and ankyrin repeats ANKK1-kinase and ANKK1-ankyrin [133]. Single nucleotide polymorphisms (SNPs) of the *ANKK1* gene are often associated with the neighboring dopamine receptor D2 (*DRD2*) gene [134]. The *TaqI A* polymorphic variant (*rs 1800497*) is located in exon 8 of the *ANKK1* gene. It was previously thought to be located in the promoter region of the *DRD2* gene. It is now known to lie more than 10 kb away from the *DRD2* gene [131,135]. The location on chromosome 11 and the structures of the *ANKK1* and *DRD2* genes are shown in Figure 3.

The *TaqI A* polymorphism in the *ANKK1* gene consists of replacing cytosine with thymine (C>T) at the restriction site for the TaqI enzyme. The amino acid substitution replaces glutamine with lysine (Glu713Lys) at locus 713 of exon 8 in the *ANKK1* gene on chromosome 11 [137]. There are two minor alleles, *T* (aka *A1*) and a *C* allele (aka *A2*). The *T* allele affects glucose metabolism in regions of the brain that have a higher D2 receptor density [138]. The occurrence of a group of *T+* genotypes (*TT* and *CT* genotypes) in the Caucasian race is observed at the level of about 31% [139,140]. An overview of association studies of *Taq1 A* polymorphism of the *ANKK1* gene in opioid addiction is presented in Table 3.

The frequency of the *T* allele is approximately 22% in the Caucasian population. Studies have shown that the presence of the *T* allele correlates with a reduced number of dopamine binding sites in the brain and a 30–40% reduction in the expression of D2 receptors in the striatum and adjacent structures (without affecting receptor affinity) [131,138,150,151,152]. According to research, the *T* allele was associated with an increased risk of alcoholism and addiction to opioids, cocaine, and tobacco [153,154,155,156,157,158]. Addicts are characterized by a lower density of dopaminergic D2 receptors in the striatum, and, therefore, signals of dopamine transfer to the cell are weaker [159]. The *TaqI A* (*T* allele) polymorphism of the *ANKK1* gene is strongly associated with a high risk of early onset of heroin use (among adolescents) and a poorer response to methadone opioid substitution treatment [160,161].

### 4.3. TagI B Polymorphism (rs 1079597) of the DRD2 Gene

Alternative splicing of the *DRD2* gene results in three isoforms of the D2 receptor that have distinct anatomical, physiological, signaling, and pharmacological properties. The following isoforms are distinguished: short presynaptic D2Sh (D2-short), long postsynaptic D2Lh (D2-long), and D2 Longer [162,163]. The *DRD2* gene plays a key role in the regulation of the mesolimbic dopaminergic pathway. Many polymorphic variants have been identified within this gene, but only a few of them have been associated with the development of opioid dependence. The *TaqI B* polymorphism (*rs 1079597*) of the *DRD2* gene is located in the first intron of the gene (1.882 bp before exon 2) and consists of the substitution of guanine with adenine (G>A). The polymorphic variant correlates with a decrease in the activity of the *DRD2* gene due to weaker binding of the dopamine D2 receptor (decreased receptor density) [138,151,152]. An overview of association studies of the *DRD2 Taq1 B* polymorphism in opioid addiction is presented in Table 4.

This polymorphism has been shown to be associated with addiction to heroin, nicotine, and cocaine, schizophrenia, and other mental and behavioral disorders [147,164,165]. The frequency of genotypes in the European population is estimated at the following levels: *GG* genotype (homozygous wild-type): 78%; *GA* genotype: 20%; and *AA* genotype: 3% [134].

### 4.4. -141C Ins/Del Polymorphism (rs 1799732) of the DRD2 Gene

The *-141C Ins/Del* polymorphism (*rs 1799732*) of the dopamine D2 receptor gene (*DRD2*) is an inertial deletion genetic variant at position 141 in the promoter region of the gene in the 5′ untranslated region (5′UTR). It consists of the insertion or deletion of a single base pair cytosine at position -141. The *Del/Del* genotype is less common in Caucasian and Chinese populations (9%) than in Japan (22%) [166]. *Del+* (*Ins/Del*, *Del/Del*) genotypes of the *-141C Ins/Del* polymorphism of the *DRD2* gene are associated with more efficient dopamine binding to the receptor and a higher density of dopamine receptors in the striatum [138]. An overview of association studies of the *-141C Ins/Del* polymorphism of the *DRD2* gene in opioid addiction is presented in Table 5.

The relationship between the *-141C Ins/Del* polymorphism of the *DRD2* gene and the development of opioid addiction and schizophrenia, an increased risk of adenoma recurrence, and poorer outcomes of antipsychotic drug therapy has been demonstrated [142,169,170].

### 4.5. A118G Polymorphism (rs 1799971) of the OPRM1 Gene

**Figure 4:**
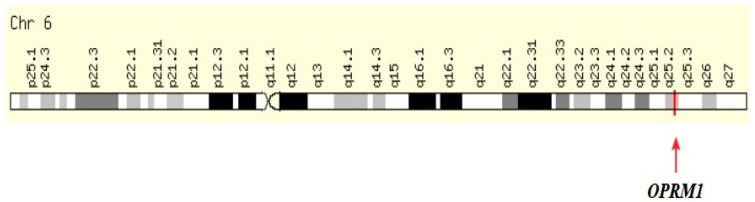
Location of the *OPRM1* gene on chromosome 6 (modified after https://www.genecards.org/cgi-bin/carddisp.pl?gene=OPRM1, Accessed date: 2 June 2024 [173]). *OPRM1* Gene-Opioid Receptor Mu 1, Protein Coding (Updated: 2 October 2024; GC06P163868; GIFtS: 61).

**Figure 5:**
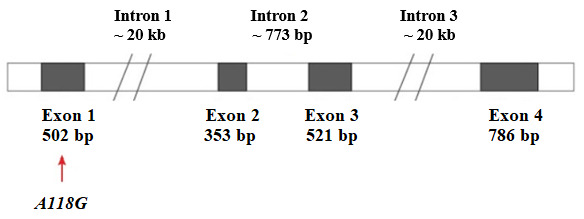
*OPRM1* gene structure and location of SNP: *A118G* (modified after Ding et al. (2013) [172]).

The human μ1 opioid receptor gene *OPRM1* is located on chromosome 6 (6q25.2), and it covers a region of 90 kb (Figure 4) [171]. In this gene, four exons are separated by three introns. Intron 2 is only 773 pb (Figure 5) [172].

The *OPRM1* gene encodes the μ1-isoform of the opioid receptor (Asp40 variant), which has three times the affinity for opioid ligands. It is the main receptor for endogenous opioid peptides and exogenous opioids, including morphine, heroin, codeine, fentanyl, buprenorphine (as a semi-synthetic derivative of the opiate alkaloid thebaine), and methadone [174,175]. Opioid receptors are involved in the regulation of reward, motivation, and addiction behaviors. The *OPRM1* gene is highly polymorphic (100% of its genetic variants have been detected). Research has confirmed that several of them are strongly associated with addiction to addictive substances or drugs. The single nucleotide polymorphism *A118G* (*rs 1799971*) in the *OPRM1* gene is located in exon 1 [176,177]. It consists of the transition of adenine to guanine at position 118, which changes the amino acid sequence by replacing asparagine with aspartic acid at position 40. The N-glycosylation site is lost, which increases the affinity for opioids and changes the half-life of the receptor in the cell membrane [34,178]. As a result of this substitution, the binding of β-endorphins to the µ1 receptor is three times stronger.

The altered binding affinity of the receptor to the opioid ligand may induce positive reinforcement, which in turn may contribute to an increased susceptibility to the development of opioid dependence. The *A118G* polymorphism is responsible for downregulation of the µ1 receptor [172,179,180]. The *G* allele adds a methylation site that reduces the level of the µ-receptor messenger RNA (mRNA), i.e., it interferes with its normal activity [34]. The frequency of the *A118G* genetic variant is highly variable. Its occurrence in the Caucasian race is observed at the level of 8–30%. In the Afro-American population, it is rare (1–3%), and in the Asian populations, it is common, even at the level of 50% [177,181,182]. The *G* allele is in the minority in many human populations (in Afro-Americans, it is at the level of 4%, in Europeans, it is ~16%, and in Asian populations, it is up to over 40% [183,184].

It should be emphasized here that association studies have determined the role of the *A118G* genetic variant of the *OPRM1* gene in the development of addiction to addictive substances or drugs, e.g., alcohol, nicotine, opioids, and gambling [185,186,187]. They have revealed the influence of this genetic variant on the effectiveness of analgesia. *G* allele carriers correlated with the need to use higher doses of opioids (morphine, oxycodone) in the treatment of cancer and postoperative pain [178,188,189]. It is suggested that *OPRM1* gene polymorphisms may affect the effectiveness of methadone treatment in opioid substitution therapy. It has been shown that the presence of the *G* allele of the *A118G* polymorphism of the *OPRM1* gene is associated with the need to take higher doses of methadone in substitution treatment [190]. The *A118G* polymorphism of the *OPRM1* gene may influence the development of opioid dependence.

The strongest association between polymorphism in the *A118G* gene and the development of opioid dependence was noted in the Asian population. Haerian and Haerian (2013) [128] analyzed 18 association studies of the *A118G* polymorphism of the *OPRM1* gene in opioid addiction. The participants were patients addicted to heroin, other opioids, and cocaine (Caucasian, Afro-American, Hispanic, and Asian populations). A relationship between the *A118G* variant and susceptibility to opioid addiction in the Asian population was observed, with the *G* allele being the predisposing factor [128]. Mistry et al. (2014) [104] confirmed that the *G* allele *rs1799971* may have a protective effect in the Hispanic population. Studies among Caucasian populations have rarely shown an association between opioid dependence and the *A118G* polymorphic variant of the *OPRM1* gene. However, the results of Bart et al. (2004) [119] revealed a significant association between the *G* allele of the *OPRM1 A118G* polymorphism and heroin addiction in central Sweden [2,119]. Schwantes-An et al. (2016) [183] conducted a meta-analysis of studies on the association of SNP A118G with addiction to opioids and other addictive substances or drugs, including nicotine, cocaine, and alcohol (9.1 addicts and 7.8 healthy people).

A significant association was found between the *A118G* variant and overall substance dependence, including a protective effect of the *G* allele of the *A118G* polymorphism on susceptibility to substance dependence [183]. Research by Ahmed et al. (2018) [129] indicate a significant relationship between the homozygous *GG* genotype and opioid addiction in the Pakistani population [129]. The relationship between the *A118G* polymorphism of the *OPRM1* gene and opioid addiction has shown ambiguous results; therefore, there is a need for further research. It has been shown that the distribution of this polymorphism varies depending on the ethnic group [130]. Having at least one copy of the *G* allele (*AG* or *GG*) of the *OPRM1* gene polymorphism is associated with a lower pain threshold and higher opioid consumption in patients during postoperative pain management. The presence of at least one copy of the *G* allele may suggest that the patient will be less sensitive to the analgesic effects of opioids and more susceptible to addiction [191,192].

It should also be taken into account that our studies [39,40,41] among opioid-dependent patients focused on the importance of genes encoding *ANKK1* serine–threonine kinase, the *DRD2* dopaminergic receptor, and the *OPRM1* µ1-opioid receptor in conditioning opioid addiction in the Polish population. The presence of the polymorphisms *TaqI A* (*rs 1800497*) of the *ANKK1* gene, *TagI B* (*rs 1079597*) of the *DRD2* gene, *-141C Ins/Del* (*rs 1799732*) of the *DRD2* gene, and *A118G* (*rs 1799971*) of the *OPRM1* gene were analyzed in patients diagnosed with opioid dependence syndrome treated with methadone substitution. At the same time, these genetic polymorphisms were tested in a group of people who met the criteria of the control group. The results of these studies regarding the *ANKK1 TaqI A* polymorphism showed a significant difference in the occurrence of the *TT* genotype in opioid-dependent patients. It was found that more people in the control group had the *CC* genotype than those in the addicted group. The analysis of the *DRD2-141C Ins/Del* polymorphism showed significant differences in the *Ins/Ins* genotype in the group of opioid addicts. It was also shown that more people from the control group had the *Ins/Del* genotype than in the group of opioid addicts. The analysis showed no differences in the frequency of *DRD2 TagI B* and *OPRM1 A118G* polymorphisms between the two groups.

Al-Eitan et al. (2021) [12] also investigated the genetic susceptibility of opioid receptor gene polymorphisms in drug addiction and analyzed candidate gene associations. They emphasized that like other complex diseases, including drug addiction, genetic factors can interfere with the disease. In their studies, three opioid genes were examined for an association with drug addiction among Jordanian males (*OPRM1*, *OPRD1*, and *OPRK1*). They propose that rs1799971 of the *OPRM1* gene is a genetic risk factor for drug addiction among Jordanian males. Their results provided additional clinical, epidemiological, and genetic knowledge that may be useful in the context of further genetic and pharmacogenetic analyses to reduce the severity of drug consumption and improve drug abstinence (Al-Eitan et al., 2021) [12]. On the other hand, Gaddis et al. (2022) [14] investigated multi-trait genome-wide associations of opioid addiction. They stated that opioid addiction (OA) is moderately heritable, yet only rs1799971, the *A118G* variant in the *OPRM1* gene, has been identified as a genome-wide significant association with OA that has been independently replicated. Gaddis et al. (2022) found that gene-based analyses identified novel genome-wide significant associations with *PPP6C* and *FURIN*. Variants within these loci appear to be pleiotropic for addiction and related traits. They observed the strongest evidence to date for the *OPRM1* gene as a lead SNP rs9478500. These genes are novel for OA; however, variants within them have been associated at genome-wide significance with related phenotypes, such as cigarette smoking, alcohol consumption, general risk taking, and schizophrenia (Gaddis et al., 2022) [14].

Based on our current studies on this subject [39,40,41], we have shown that the homozygous *TT* mutant of the *TaqI A* polymorphism (*rs 1800497*) in the *ANKK1* gene may be a factor for increased risk of opioid addiction in the Polish population. In the group of people addicted to opioids, the frequency of the *TT* genotype was 48%, the frequency of the group of *T+* genotypes (*TT* and *CT*) was 85%, and the frequency of *T* alleles was 66.4%. In the control group, the frequency of the *TT* genotype was estimated at 12%, the frequency of the group of *T+* genotypes (*TT* and *CT*) was 42%, and the frequency of *T* alleles was 26.7%. In the control, the incidence of homozygous wild-type *CC* was 58.6% (and it was 15% in the group of patients). The frequency of *T+* genotype groups (*TT* and *CT* genotypes) in Caucasians in healthy individuals is 31%, and the frequency of *T* alleles in Caucasians in healthy individuals is 22% [139,140]. *ANKK1 TaqI A* polymorphism is believed to be strongly associated with a high risk of early onset of heroin use in adolescents and a poorer response to methadone opioid substitution treatment [160,161]. The *T* allele is primarily associated with an increased risk of alcoholism but also addiction to opioids, cocaine, and tobacco [153,154,155,156,157,158]. The *T* allele affects glucose metabolism in regions of the brain with higher dopaminergic D2 receptor density [138]. Studies have shown that the presence of the *T* allele correlates with a reduced number of dopamine binding sites in the brain and a 30–40% reduction in the expression of D2 receptors in the striatum and adjacent structures (without affecting receptor affinity) [131,138,150,151,152]. Addicts are characterized by a lower density of dopaminergic D2 receptors in the striatum, and, therefore, the signals of dopamine transfer to the cell are weaker, which is why these people feel less satisfied and happy [159]. Our research on this subject [39,40,41] showed that more patients addicted to opioids had a *TT* genotype (48%) than the controls (12%).

Currently, much attention is focused on the analysis of the relationships among changes in genetic material under the influences of environmental stressors, among which the most important are the place of residence (the type of environment and the so-called catchment area), diet, addictions, occupational exposure, hereditary diseases, and genetic conditions in the family. The latest studies currently available draw attention to the priority of these factors as direct sources of so-called environmental diseases. For example, Khan et al. (2024) [10] analyzed the association of the genetic polymorphism of glutathione S-transferases (GSTs) with colorectal cancer susceptibility in snuff (Naswar) addicts. Their findings suggest that *GSTM1* and *GSTT1* polymorphism and its combination with *GSTP1* may be associated with colorectal cancer (CRC) susceptibility in the Naswar-addicted Pashtun population of Pakistan. These authors conclude that *GSTM1* null and *GSTT1* null genotypes individually significantly contributed to the risk of cancer. The combined *GSTM1* null and *GSTT1* null led to a significant risk; similarly, the combined *GSTM1* null and *GSTP1 Ile*/*Val* or *Val*/*Val* genotypes as well as the *GSTT1* null and *GSTP1 Ile*/*Val* or *Val*/*Val* genotypes significantly increased the individuals’ susceptibility to cancer. The combination of three *GST* genotypes, i.e., *GSTM1* null, *GSTT1* null, and *GSTP1 Ile*/*Val* or *Val*/*Val* genotypes, also demonstrated gene–gene interaction and further contributed to the increased risk of colorectal cancer. Khan et al. (2024) [10] found that the presence of *GST* null genotypes is associated with CRC risk because the null or missing genotype cannot detoxify the tobacco carcinogens. These authors suggest that authorities should take strict measures to ban/discourage the use of Naswar (snuff) and other forms of tobacco to control tobacco-related cancers (Khan et al., 2024) [10].

Also, Parker et al. (2024) [22] showed their findings, which indicate shared genetic underpinnings for the cortical brain structure and blood immune markers, with implications for neurodevelopment and understanding the etiology of brain-related disorders. They found that genetic overlap between the cortical structure and the immune markers exhibited mixed effect directions that probably represent the complex relationship between the brain and the immune system. Biological underpinnings implicate neural cell types that may mediate these associations. They discovered a consistent enrichment for genes associated with schizophrenia, a disorder with neurodevelopmental origins that has been linked to alterations in the cortical brain structure and the immune system. These findings inform potential mechanisms underlying the relationship between the brain and blood immune markers, with important implications for brain development and the etiology of brain-related disorders (Parker et al., 2024) [22].

Another aspect of the genetic determinants of addiction was studied by Levey and Gelernter (2023) [13], who analyzed a multi-ancestry, genome-wide association study of cannabis use disorder (CanUD), which yielded insight into disease biology and public health implications. They suggest that genetically informed causal relationship analysis indicated a possible effect of genetic liability for CanUD on lung cancer risk, suggesting potential unanticipated future medical and psychiatric public health consequences. They state that this requires further study to disentangle these findings from other known risk factors, such as cigarette smoking. The authors identify a clear difference between cannabis use and CanUD, with genetic liability for CanUD being much more closely associated with psychopathology and disability. They found greater heritability enrichment in fetal than adult brain tissue, supporting an important role of development in laying the biological basis for CanUD (Levey, Gelernter 2023) [13].

Also, according to Montalban et al. (2023) [17], different experiments’ evidence highlights the importance of genetic variants in the development of psychiatric and metabolic conditions. Among these, the *TaqIA* polymorphism is one of the most commonly studied in psychiatry. *TaqIA* is located in the gene that codes for the ankyrin repeat and kinase domain containing 1 kinase (Ankk1) near the dopamine D2 receptor (D2R). Homozygous expression of the *A1* allele correlates with a 30% to 40% reduction of striatal D2R, a typical feature of addiction, overeating, and other psychiatric pathologies. The mechanisms by which the variant influences dopamine signaling and behavior are unknown. Research by Montalban et al. (2023) demonstrated that the *Ankk1* gene is necessary for the integrity of striatal functions, and they reveal a new role for Ankk1 in the regulation of body metabolism. These studies provide the first reverse translational approach exploring the biological functions of Ankk1 in the central regulation of both the metabolic and the reward functions, and they further translate the metabolic phenotype discovered in mice to humans. These results show that Ankk1 loss of function is sufficient to mimic some of the phenotypic characteristics of *Taq1A* individuals, and they point toward Ankk1 as a potential molecular hub connecting striatal D2R-SPNs to the control of energy homeostasis (Montalban et al., 2023) [17].

In the mutual relationships between the actions of ions of chemical compounds and in the reactions generated by enzymatic and non-enzymatic compounds and by heat shock proteins (HSPs) participating in pro-antioxidative reactions and lipoperoxidation, in which gene polymorphisms are the causative factor, the forms of these polymorphisms play a significant role. The factor predisposing to the development of opioid addiction may be a reduced number of dopamine binding sites in the brain and the reduced expression and density of dopaminergic D2 receptors in the striatum and adjacent structures (presence of *T* allele). Association studies have shown the role of the *TaqI A* genetic variant of the *ANKK1* gene in the development of opioid addiction as a result of the modulation of the dopaminergic system (Lawford et al., 2000 [141], Shahmoradgoli et al., 2005 [143], Perez de los Cobos et al., 2007 [144]). Similar genetic studies were conducted on the Polish population by Chmielowiec and Boroń (2020) [193] and by Masiak et al. (2020) [194], but they concerned people addicted to addictive substances or drugs [193,194]. Lawford et al. (2000) [141] showed a significantly higher frequency of the *T* allele in heroin addicts compared to controls in the Australian population. They also linked a poorer response to methadone treatment with carrying the *T* allele [141]. In the studies by Shahmoradgoli et al. (2005) [143], a higher frequency of the *T* allele and the *TT* genotype in Iraqi opium addicts compared to controls was discussed. Perez de los Cobos et al. (2007) [144], in research on the Hispanic population, showed significant differences in the frequency of the *TT* genotype between heroin addicts and the controls regardless of gender and significant differences in the frequency of the *T* allele between heroin addicts and the controls, but only in men. Higher frequencies of the *T* allele and the *TT* genotype have been found in heroin addicts [144]. Hou and Li (2009) [146] reported a higher frequency of the *T* allele in heroin addicts compared to controls in the Chinese population (prone to heroin abuse in dominance or codominance models).

Considering the effects of these mutual dependencies of environmental causative sources and epigenetic factors generating genetic changes (gene polymorphisms), attention is drawn to the importance of biochemical processes leading to changes in the directions of metabolic pathways. This takes place at various levels of organization (subcellular, cellular, organ, etc.). As a result, the efficiency of natural defense mechanisms (enzymatic, non-enzymatic, heat shock proteins) is inhibited and/or they slowly disappear. Physiological failures, organ dysfunctions, and changes in the body’s condition occur. As a result of all of these slow but systematic processes, pathophysiological and histopathological changes develop in various organs, especially the weakest ones. Such effects are noted by, among others, Huang et al. (2024) [16], who investigated the changes in the neurofilament light chain (NFL) in alcohol-dependent patients after withdrawal and analyzed the genetic effect of the *ALDH2* polymorphism. These authors concluded that the plasma of the NFL level was increased in patients with alcohol dependence (AD) and reduced after early abstinence. Findings by Huang et al. (2024) demonstrated the blood NFL levels were higher in patients with AD than healthy controls, and this correlated well with the severity of AD, craving, and drinking-related biochemistry markers. Reduction of the NFL level corroborated well with the improvement of clinical symptoms. The *ALDH2* rs671 polymorphism may play a role in modulating the extent of neuroaxonal injury and its recovery (Huang et al., 2024). They found that AD patients with the *ALDH2 GA* genotype tend to have a more severe neurotoxicity. This finding may indicate a need to genotype AD patients for *ALDH2* when using blood NFL as a potential neurotoxic indicator for clinical diagnosis, evaluation of alcohol-induced neuroaxonal injury, and monitoring of treatment outcomes. Also, given that the common phenotype of the *GA* genotype is facial flushing following alcohol consumption, it is possible that individuals with this experience might suffer from more severe neuroaxonal injury (Huang et al., 2024) [16].

Also, Mufford et al. (2024) [18] emphasize that whereas genetic variants influencing total amygdala volume have been identified, the genetic architecture of its distinct nuclei has yet to be explored. In their studies of the genetic architecture of amygdala nuclei, Mufford et al. (2024) addressed the issue of whether increased phenotypic specificity through nuclei segmentation aids genetic discoverability and elucidates the extent of shared genetic architecture and biological pathways with related disorders. These authors demonstrated that through investigation of amygdala nuclei volumes, they have identified novel candidate loci in the neurobiology of amygdala volume. These nuclei volumes have unique associations with biological pathways and genetic overlap with psychiatric disorders (Mufford et al., 2024). Their findings indicate that the amygdala nuclei have specific associations with biological processes and genetic overlap with brain disorders. However, continued studies are needed to further our understanding of the genes implicated in the genetic architecture of amygdala nuclei [18].

Important studies investigating these issues have examined the role of changes in genetic material in shaping disorders and relapses associated with the use of psychoactive substances or opioids. Thus, Strońska-Pluta et al. (2024) [19] analyzed the relationship between the brain-derived neurotrophic factor gene polymorphism (*Val66Met*) and substance use disorder and relapse. The results of these studies provide further evidence that personality traits, anxiety, and the rs6265 polymorphism of the *BDNF* gene may be risk factors for susceptibility to addiction to psychoactive substances. They can be a predictor of addiction relapse, but further extensive studies are required to confirm these findings. It should be emphasized that the analysis of genotypes and alleles in relation to personality factors is justified. However, it should be remembered that this may also be a factor limiting the interpretation of the study. These studies require the creation of homogeneous subgroups, including those that take into account the personality traits of the studied individuals (Strońska-Pluta et al., 2024) [19].

This aspect was also analyzed in research by Sprague et al. (2024) [15], who evaluated the influence of genetic variants related to opioid use disorder (OUD) using multiple logistic regression analysis in self-reported assigned African American/Afro-Caribbean and European biogeographical ancestry groups (BGAGs) and by sex. Their findings suggest that variant testing relative to OUD risk can be applied across BGAGs and sex; however, studies in larger populations are needed (Sprague et al., 2024 [15]). These authors stated that biogeographical genetic ancestry group designation of African American/Afro-Caribbean or European populations relative to rs2740574 and rs324029 did not influence either variant’s impact on OUD risk. Similarly, sex did not influence the impact of rs15524, rs776746, rs2740574, rs324029, rs2654754, or rs2069514 on OUD risk. These findings suggest that the evaluation of these variants can be utilized to gain insight relative to OUD risk across sexes in African American/Afro-Caribbean or European individuals, although further work needs to be done in larger populations of these patients (Sprague et al., 2024) [15]. Findings by Sprague et al. (2024) [15] suggest that variant testing relative to OUD risk can be applied across BGAGs and sex; however, studies in larger populations are needed [15].

On the other hand, Zhang et al. (2019) [11] showed that the fatty acid amide hydrolase (FAAH) gene was involved in the modulation of reward and addiction pathophysiology of illicit drugs abuse, and its polymorphisms might be associated with risk of methamphetamine (METH) dependence. Their data indicate that the FAAH may play an important role in the pathophysiological process of METH dependence, and the 385C/A polymorphism may be associated with METH dependence susceptibility in Chinese populations (Zhang et al., 2019) [11]. Also, Ferraguti et al. (2024) [21] analyzed the genetic aspect of the role of DNA sequence variations affecting the serotonin transporter. They found significant differences in the allelic and genotypic frequencies of the tri-allelic polymorphism, with higher-function alleles and genotypes more represented in the control population. They conclude that transcriptional regulation and activity have an impact on alcohol addiction. Their results obtained for the tri-allelic polymorphism in alcohol dependence confirm what is already present in part of the literature. The role of haplotypes requires further studies to be clarified (Ferraguti et al., 2024) [21].

Specific aspects of the determinants of addiction and changes in genetic material were studied in detail by Guerin et al. (2024) [20]. They showed the associations between methamphetamine use disorder and *SLC18A1*, *SLC18A2*, *BDNF*, and *FAAH* gene sequence variants and expression levels. According to their results, *SLC18A1* was identified for the first time as playing a potential role in methamphetamine use disorders. Both *SLC18A2* and *FAAH* blood mRNA levels were lower in people who used methamphetamine relative to controls, with higher *SLC18A2* levels associated with better cognitive flexibility, whereas lower *FAAH* expression was associated with better inhibitory control in people who used methamphetamine. Lower levels of blood *SLC18A2* and *FAAH* mRNA in people with methamphetamine use disorder suggest reduced monoamine reuptake, recycling, or release and higher anandamide levels in this clinical group, which may be potential therapeutic targets. They found a potential role of the *SLC18A1* Pro4Thr variant in conferring a risk for methamphetamine use disorders (Guerin et al., 2024) [20]. These results can inform larger hypothesis-driven clinical and preclinical studies to further characterize the contribution of these genes to the development of methamphetamine use disorders [20]. Meanwhile, Xie et al. (2021) [9] reported an association between γ-aminobutyric acid (GABA) receptor delta subunit gene polymorphisms and heroin addiction. Their results suggest that *GABRD* rs13303344 may contribute to susceptibility to heroin addiction, and it is associated with the drug cravings of heroin-dependent patients. The GABA system may be a suitable pharmacotherapeutic target for the treatment of drug addiction, and variants of this system may also affect the response to treatment (Xie et al., 2021) [9].

Different results were reported by Vereczkei et al. (2013) [147] and Cai et al. (2015) [148]. Vereczkei et al. (2013) [147] showed in Hungarian populations significant differences in the frequency of genotypes between heroin addicts treated with methadone and in the control group. They found that in both heroin addicts and healthy subjects the *CC* genotype was dominant (addicts 60%; control 69%) and the *TT* genotype was in the minority (addicts 6%; control 3%) [147]. Cai et al. (2015) [148] reported a higher frequency of the *C* allele and the *CC* genotype in Chinese populations in people addicted to opioids with post nasal drip syndrome (PND) compared to the control group [148]. In the studies by Li et al. (2002) [142] on Chinese populations, Crettol et al. (2008) [145] on Swiss populations, and Yilbas et al. (2016) [149] on the Turkish population, no significant differences in the frequency of the *ANKK1 TaqI A* polymorphism alleles/genotypes were found between heroin and other opioid addicts and healthy individuals.

In our research on this subject [39,40,41], the analysis of the frequency of occurrence of *DRD2-141C Ins/Del* polymorphism genotypes showed that 100% of genotypes in the group of opioid addicts are homozygous *Ins/Ins* wild-type (93% in the control). It was found that the most common genotype was the *Ins/Del* heterozygous mutant (frequencies of 7% in the controls and 0% in the patients). The *Del/Del* genotype was not found in the control group or in the group of people addicted to opioids. *Del+* (*Ins/Del*, *Del/Del*) genotypes of the *DRD2-141C Ins/Del* polymorphism are associated with more efficient dopamine binding to the receptor and a higher density of dopaminergic receptors in the striatum [138]. The relationship between the *DRD2-141C Ins/Del* polymorphism and the development of opioid addiction and schizophrenia, increased risk of adenoma recurrence, and poorer outcomes of antipsychotic drug therapy has been demonstrated [142,169,170]. In our studies [39,40,41], 100% of genotypes found in people addicted to opioids were homozygous wild-type *Ins/Ins* (without *Del* allele); therefore, a factor predisposing to the development of opioid addiction may be less effective binding of dopamine to the dopaminergic D2 receptor and a lower density of dopaminergic receptors in the striatum. Few candidate gene association studies have revealed the importance of *DRD2 -141C Ins/Del* polymorphism in the development of opioid use disorder or addiction syndrome.

Very few studies were found that concerned Asian populations, and one of them additionally concerned Caucasian (German) populations. Li et al. (2002) [142] conducted research on 121 heroin addicts and 194 healthy people (Chinese population), and a significant difference was found in the frequency of the *Ins/Ins* genotype. The *Ins/Ins* homozygous mutation was observed more often in the people addicted to opioids [165]. Xu et al. (2004) [167] showed that the *Del* allele plays a minor role in heroin addiction in Chinese populations and that it plays no role in heroin addiction in German populations. Studies by Shao et al. (2005) [168] on Chinese populations showed no significant differences in the frequency of alleles/genotypes of the *DRD2-141C Ins/Del* polymorphism between heroin addicts and the controls. Al-Eitan et al. (2012b) [116] showed that the *Del* allele plays a significant role in heroin addiction in Jordanian populations. Li et al. (1998) [166] report that the *Del/Del* genotype is less common in Caucasian and Chinese populations (9%) than in Japanese populations (22%). In our research on this subject [39,41], the *Del/Del* genotype was not observed in the people addicted to opioids or in the controls.

We conducted analyses of variance to examine differences between healthy and sick people in the interaction of the following studied factors: gene polymorphisms (*ANKK1 TaqI A* genotype *CT*, *DRD2 TaqI B* genotype *GA*, *DRD2 TaqI B* genotype *AA*), consumption of vegetables, consumption of dairy products, work on harmful factors and their impact on the levels of chemical elements, enzymatic and non-enzymatic factors, and lipid peroxidation [39,40,41,42]. It should be emphasized that these are the first studies of this type including patients addicted to addictive substances. Thus, we showed the following.

(1) Control subjects with *ANKK1 TaqI A-CC* and *TT* genotypes had lower concentrations of Mn and Fe in the plasma compared to those addicted to opioids. Opioid-dependent individuals with *ANKK1 TaqI A-CT*, *CC*, and *TT* genotypes had higher plasma Mn and Fe concentrations than controls. Opioid addicts with the *ANKK1 TaqI A-CT* genotype had higher concentrations of Mn and Fe in the plasma compared to addicts with *CC* and *TT* genotypes. Control subjects with *ANKK1 TaqI A-CC* and *TT* genotypes had significantly lower plasma Yb concentrations than control subjects with *CT* genotypes and addicts with *CC* and *TT* genotypes.
(2) Control subjects with *DRD2 TaqI B-GG* and *AA* genotypes had lower plasma Tl levels than addicted subjects. Opioid-dependent individuals with *DRD2 TaqI B-GA*, *GG*, and *AA* genotypes had higher plasma Tl concentrations compared to controls with these genotypes. Opioid addicts with the *DRD2 TaqI B-GA* genotype had higher plasma Tl levels than those with *GG* and *AA* genotypes. Opioid-dependent individuals with *DRD2 TaqI B-GA*, *GG*, and *AA* genotypes had higher plasma Tl concentrations compared to controls with these genotypes. Opioid addicts with the *DRD2 TaqI B-AA* genotype had lower Tl levels than those with *GA* and *GG* genotypes.
(3) Patients addicted to addictive substances who consumed vegetables and who did not consume them had lower concentrations of Lu compared to controls who consumed vegetables and who did not consume vegetables. Controls who consumed vegetables had higher plasma Lu concentrations than those who did not consume vegetables. Consumed vegetables could be a source of lutetium in the plasma of control and addicted people.

According to our latest and continuing research [39,40,41,42], it turned out that in the mutual relationships generated by enzymatic and non-enzymatic compounds participating in pro-antioxidant reactions and lipoperoxidation processes, in which gene polymorphisms are the first causative factor, rare earth ions also play a significant role. Rare earth elements, including lutetium, are found in low concentrations in the soil, plants, and the atmosphere [195]. They can also accumulate in the environment as a result of anthropogenic activities (mining and artificial fertilizers enriched with rare earth elements) and persist in it due to low mobility. Chronic exposure to higher concentrations of these metals can have serious consequences for ecosystems, groundwater, agricultural production, and human health [196]. It was shown that people in the control group had higher plasma Lu levels than those addicted to opioids. People from the controls (58%) consumed vegetables more often than people addicted to opioids (34%) [39,40,41]. Addicts who consumed dairy and who did not consume it had significantly higher plasma Ca concentrations than those from the control who consumed dairy and who did not consume it. Opioid addicts who did not consume dairy had higher plasma Sc levels than addicts who consumed dairy and dairy-free and non-dairy controls. Opioid addicts who consumed dairy products had higher Sc levels than those from the control who did not consume dairy products [39,40,41]. Addictive substances and other determinants of addiction, which generate disorders associated with their use, in connection with the lack of dairy products, cause significantly higher concentrations of Sb in addicted people who used them. Opioid-dependent individuals who consumed dairy had higher Sb concentrations compared to those from the control who consumed dairy and those who did not consume dairy. Addicts who were exposed to harmful factors and those who were not exposed had significantly lower plasma P concentrations compared to control subjects who were not exposed to harmful factors. Dependent patients not exposed to harmful factors had lower plasma P concentrations compared to the controls [39,40,41].

In summary, knowledge about the determinants of addiction (SUD) to addictive substances or drugs is currently poor and requires supplementing. People are susceptible to addiction (SUD) to varying degrees. Based on the results of our research [39,40,41,42], it was shown that the strongest factors predisposing people to the occurrence and development of addiction are male sex, place of residence in large cities, employment (type of work), and drug and addiction in the family; destabilization of the elemental economy was also demonstrated in the patients. Significantly lower P concentrations and higher concentrations of Na, K, and Ca in the plasma of people addicted to addictive substances compared to the controls were found, which may be the result of changes in the metabolism of these elements under the influence of addiction. The increase in the concentration of microelements, toxic metals (Cd, Hg, As, Pb), and rare earth elements (Sc, La, Ce, Pr, Eu, Gd, Nd) may indicate their participation in the development of opioid addiction. Taking into account the mechanisms of action of chemical elements and data from the literature, it can be concluded that Cd, Pb, Ni, and Mn may have the most important effect on the increasing susceptibility to the development of addiction. Interactions shaped by chemical elements may play an important role in the development of disorders associated with the use of addictive substances; therefore, elements that show the most correlations in patients may be factors in the development of addiction (Pr, Na, Mn, Y, Sc, La, Cr, Al, Ca, and Sb). We have also shown that Hg may have a smaller share in the development of addiction [39,40,41,42].

Our studies confirmed that the *ANKK1 TagI A-TT* and *DRD2-141C Ins/Del-Ins/Ins* genotypes may be potential genetic markers that increase susceptibility to opioid addiction in the Polish population. It was found that in opioid-dependent people with the *ANKK1 TaqI A-TT* genotype, concentrations of Na, Al, Fe, Mn, Y, La, Ce, Pr, and Tb were significantly lower than in people with the *CT* and *CC* genotypes [39,40,41]. The share of genetic factors in susceptibility to the development of drug addiction is 40–60%, and modern progress in genetics allows for the identification of specific variants that may predispose an individual to these disorders. Twin and family studies have shown that there are critical genetic and environmental components in the inheritance of SUD [101,102,103,118,119,120].

## 5. Conclusions

Innovative aspects in terms of missing data regarding the relationships between gene polymorphisms correlated with addiction (SUD) and chemical elements are analyzed on the basis of previous rare research, as well as our current studies. Based on these analyses, the importance of these interrelationships between DNA-level variability and physiological responses to environmental stressors can be inferred.

The innovative element of this review is the demonstration of relationships between addiction, environmental and genetic factors (gene polymorphism), and toxic metals, which may indicate a potential role of epigenetic mechanisms in the development of addiction. Toxic ions are factors that change epigenetic modifications, which contribute to the over-expression of genes, especially promoters of inflammation-related gene polymorphisms, which ultimately contributes to the over-expression of genes and the promotion of increased susceptibility to addiction.

Another innovative aspect of this review is the demonstration that the homozygous *TT* mutant of the *ANKK1 TaqI A* polymorphism *rs 1800497* may be a factor in increased risk of opioid dependence. A factor predisposing individuals with the *TT* (*Ins/Ins*) genotype to the development of addiction and opioid dependence may be less effective dopamine binding and reduced expression and density of dopaminergic D2 receptors in the striatum and adjacent structures. We discuss that in addicted patients with the *ANKK1 TaqI A-TT* genotype, concentrations of Na, Al, Fe, Mn, Y, La, Ce, Pr, and Tb are lower than in those with the *CT* and *CC* genotypes. Our review shows that the homozygous *Ins/Ins* wild-type *DRD2-141C Ins*/*Del* polymorphism *rs 1799732* may be a risk factor for opioid dependence.

Psychoactive-dependent patients are at high risk for disrupting homeostatic processes. We can observe that ionic correlations and environmental and genetic factors may be related and may potentially contribute to addiction. Thus, increases in the concentrations of microelements (Mn, Fe, Cu, Co, Zn, Cr, Ni, Tl, Se, Al, B, Mo, V, Sn, Sb, Ag, Sr, and Ba), toxic metals (Cd, Hg, As, and Pb), and rare earth elements (Sc, La, Ce, Pr, Eu, Gd, and Nd) (with the greatest correlations with Na, Mn, Cr, Al, Ca, Sb, Cd, Pb, As, Hg, and Ni) may be factors shaping the development of addiction. Taking into account the mechanisms of toxic effects of these elements and data from the literature, it can be concluded that Cd, Pb, As, Hg, Al, Ni, and Mn may have the most important influence on increasing susceptibility to the development of addiction.

Interactions between environmental and genetic factors, especially *ANKK1* (*TaqI A*), *ANKK1* (*Taq1 A-CT*), *DRD2* (*TaqI B*, *DRD2 Taq1 B-GA*, *DRD2 Taq1 B-AA*, *DRD2-141C Ins/Del*), and *OPRM1* (*A118G*), in patients addicted to addictive substances or drugs and their relationships with physiological responses confirm the importance of internal factors as determinants of addiction. They are significantly varied when taking into account gender and region, and they may contribute to increased susceptibility to opioid addiction.

## Figures and Tables

**Table 1:** Hypotheses related to the interaction of Zn, Cd, Cr, and Pb with morphine in the central nervous system (modified after Kupnicka et al. (2020) [52]).

Exposure to Elements	Interaction with Morphine	Mechanism of Interaction
Zn	- Reduction of severity of morphine dependence	- Interaction with groups of opioid SH receptors, which affects the number of receptors and their affinity - Inhibition of μ-receptor agonists’ binding - antagonistic action on NMDA receptor
Cd	- Increased morphine sensitization	- Dysregulation of dopamine D2 receptor function - Antagonistic action on NMDA receptor
Pb	- Increased morphine tolerance - Increased worsening of withdrawal symptoms	- Effects on dopamine metabolism - Increased expression of the D2 receptor in the brain
	- Neuroinflammation - Increased morphine tolerance	- Glial activation - Increased expression of purinergic receptors P2x4, P2x7 - Increased expression of adenosine A1 receptor
Cr	- Reduction of severity of morphine dependence - Reduction of withdrawal symptoms - Conditioned place preference stimulation	- Increased levels of serotonin, dopamine, and norepinephrine

**Table 2:** Genetic Addiction Risk Score (GARS) panel (modified after Blum et al. (2014) [124]).

Gene	Risk Alleles	Prime Function
dopamine D1 receptor *DRD1*	*48G*	Regulation of dopamine release in the nucleus accumbens
dopamine D2 receptor *ANKK1/DRD2*	*Taq I A1*	Controls synthesis of dopamine D2 receptors
dopamine D3 receptor *DRD3*	*C*	Carriers sensitive to cocaine, opioids, alcohol, and nicotine
dopamine D4 receptor *DRD4*	*7R*	predisposed to novelty seeking and ADHD
dopamine active transporter *DAT1*	*9R*	Fast transport of synaptic dopamine back into pre-neuron, leading to hypodopaminergic trait
serotonin transporter *SLC6A4 (5-HTTLPR)*	*S*	Fast transport of serotonin back into neuron
mu-opioid receptor *OPRM1*	*G*	Predisposes to heroin addiction and pain sensitivity
GABA B3 receptor *GABRB3*	*181*	Predisposes to anxiety disorders
monoamine oxidase A *MAOA*	*3.5R*, *4R*, *5R*	Fast catabolism of mitochondria dopamine
catechol-o-methyltransferase *COMT*	*G*	Val substitution leads to fast catabolism of synaptic dopamine, leading to reward deficiency syndrome

**Table 3:** Association of *Taq1 A* polymorphism of *ANKK1* gene in opioid addiction (SUD).

*Taq1 A* (*rs 1800497*)		
Research Results—Influence on Opioid Dependence	Population	Reference
- Higher frequency of T allele in heroin addicts compared to the control group - Weaker response to methadone treatment associated with T allele carriers	Caucasian/ Australian	(Lawford et al., 2000) [141]
- No significant differences in allele/genotype frequencies between heroin addicts and controls	Asian/ Chinese	(Li et al., 2002) [142]
- Higher frequency of T allele in opium addicts compared to the control group - Higher frequency of TT genotype in opium addicts compared to the control group	Caucasian/ Iranian	(Shahmoradgoli et al., 2005) [143]
- Significant differences in the prevalence of the TT genotype between heroin addicts and controls (regardless of gender) - Significant differences in the frequency of the T allele between heroin addicts and controls (in men)	Latina	(Perez de los Cobos et al., 2007) [144]
- No significant differences in the frequency of alleles between opioid-dependent people treated with methadone and in the control group (healthy people)	Caucasian/ Swiss	(Crettol et al., 2008) [145]
- Higher frequency of T allele (TT and CT genotypes) in heroin addicts compared to controls (prone to heroin abuse in dominance or codominance models)	Asian/ Chinese	(Hou, Li 2009) [146]
- Higher prevalence of CC genotype in opioid-dependent individuals	Caucasian/ Hungarian	(Vereczkei et al., 2013) [147]
- Higher frequency of the CC genotype in opioid-dependent people with post nasal drip syndrome (PND) compared to the control group - Higher frequency of the C allele in opioid-dependent people with post nasal drip syndrome (PND) compared to the control group	Asian/ Chinese	(Cai et al., 2015) [148]
- No significant differences in the frequency of the T allele between heroin addicts and the control group	Caucasian/ Asian/ Turkish	(Yilbas et al., 2016) [149]

**Table 4:** Association of the *Taq1 B* polymorphism of the *DRD2* gene in opioid addiction (SUD).

*Taq1 B* (*rs 1079597*)		
Research Results—Influence on Opioid Dependence	Population	Reference
- Significant differences in the frequency of polymorphism genotypes between heroin addicts treated with methadone and in the control group	Caucasian/ Hungarian	(Vereczkei et al., 2013) [147]

**Table 5:** Association of the *-141C Ins/Del* polymorphism of the *DRD2* gene in opioid addiction (SUD).

*-141C Ins/Del* (*rs 1799732*)		
Research Results—Influence on Opioid Dependence	Population	Reference
- The *Ins/Ins* genotype was more common in heroin addicts	Asian/ Chinese	(Li et al., 2002) [142]
- The *Del* allele plays a minor role in heroin addiction in the Chinese population and no role in the German population	Asian/ Chinese Caucasian/ German	(Xu et al., 2004) [167]
- No significant differences in the frequency of polymorphism alleles/genotypes between heroin addicts and the control group	Asian/ Chinese	(Shao et al., 2005) [168]
- The *Del* allele plays a significant role in heroin addiction in the Jordanian population	Asian/ Jordanian	(Al-Eitan et al., 2012b) [116]

## Abbreviations

A	adenine
*A118G*	single nucleotide polymorphism of the gene *OPRM1* (*rs 1799971*:A>G)
AD	alcohol dependence
ADHD	Attention-deficit hyperactivity disorder
Ag	silver
Al	aluminum
AMPA	AMPA-type glutamate receptor selectively activated by α-amino-3-hydroxy-5-methyl-4-isoxazolepropionic acid
*ANKK1*	ankyrin repeat and kinase domain controlling 1 (kinase domain containing 1 kinase)
As	arsenic
B	boron
BGAGs	biogeographical ancestry groups
bp	base pairs
C	cytosine
cAMP	cyclic adenosine monophosphate
CanUD	cannabis use disorder
Cd	cadmium
Ce	cerium
Co	cobalt
*COMT*	gene encoding catechol-O-methytransferase
CpG (CpG islands)	unmethylated DNA segments characteristic of vertebrate gene promoters
CPP	conditioned place preference
Cr	chromium
CRC	colorectal cancer
Cu	copper
D1	dopamine receptor D1
D2	dopamine receptor D2
D2Lh	long isoform of the receptor D2
D2R	dopamine D2 receptor
D2Sh	short isoform of the receptor D2
D3	dopamine receptor D3
D4	dopamine receptor D4
D5	dopamine receptor D5
DAT1	dopamine active transporter
DβH	dopamine β-hydroxylase
*DRD1*	D1 receptor encoding gene
*DRD2*	D2 receptor encoding gene
*DRD4*	D4 receptor encoding gene
EMCDDA	European Monitoring Center for Drugs and Drug Addiction
Eu	europium
FDA	U.S. Food and Drug Administration
Fe	iron
G	guanine
GABA	γ-aminobutyric acid
GARS	Genetic Addiction Risk Score
Gd	gadolinium
GSTs	glutathione S-transferases
Hg	mercury
HSPs	heat shock proteins
5-HTTLPR	serotonin transporter
ICD	International Statistical Classification of Diseases and Related Health Problems
kb	kilo base pairs
La	lanthanum
µ (MOP)	mu opioid receptor
MAO	monoamine oxidase
MDA	malondialdehyde
METH	methamphetamine
miRNA	micro-RNA
Mn	manganese
Mo	molybdenum
NAc	nucleus accumbens
Nd	neodymium
NFL	neurofilament light chain
Ni	nickel
NMDA	N-methyl-D-aspartate receptor
OA	opioid addiction
*OPRM1*	μ1 opioid receptor encoding gene
OR	odds ratio
OUD	opioid use disorder
P2X	P2X purinergic receptors
Pb	lead
PND	post nasal drip syndrome
Pr	praxeodymium
RIP	receptor-interacting protein
Sb	antimony
Sc	scandium
Se	selenium
Sn	tin
SNP	single nucleotide polymorphism
SOD	superoxide dismutase
Sr	strontium
SUD	substance use disorder
ROS	reactive oxygen species
T	thymine
*TaqI*	restriction enzyme
*Taq1 A*	single nucleotide polymorphism of the gene *ANKK1* (*rs 1800497*:C>T)
*Taq1 B*	single nucleotide polymorphism of the gene *DRD2* (*rs 1079597*:G>A)
Tl	thallium
V	vanadium
VTA	ventral tegmental area
WHO	World Health Organization
*-141C Ins/Del*	single nucleotide polymorphism of the gene *DRD2* (*rs 1799732*:C>Del)
5′UTR	5′ untranslated region
Zn	zinc
